# Diagnostic and therapeutic challenges in primary vitreoretinal lymphoma: a practical clinical approach

**DOI:** 10.22336/rjo.2026.29

**Published:** 2026

**Authors:** Mihai Luca Cioboată, Ioana Tofolean, Suher Abduraman, Bogdana Maliș, Radu Burcea, Miruna Cioboată

**Affiliations:** 1 “Prof. Dr. Mircea Olteanu” Clinical Institute of Ophthalmological Emergencies, Bucharest, Romania; 2 “Carol Davila” University of Medicine and Pharmacy, Bucharest, Romania

**Keywords:** primary vitreoretinal lymphoma, intraocular lymphoma, vitrectomy biopsy, MYD88 mutation, intravitreal methotrexate, PVRL = primary vitreoretinal lymphoma, PCNSL = primary central nervous system lymphoma, CNS = central nervous system, OCT = optical coherence tomography, FAF = fundus autofluorescence, FA = fluorescein angiography, ICGA = indocyanine green angiography, CSF = cerebrospinal fluid, CT = computed tomography, MRI = magnetic resonance imaging, MTX = Methotrexate, BTK = Bruton’s tyrosine kinase, IgH = immunoglobulin heavy chain

## Abstract

**Background:**

Primary vitreoretinal lymphoma (PVRL) is a rare but aggressive subtype of extranodal diffuse large B-cell lymphoma and is closely related to primary central nervous system lymphoma (PCNSL). Its ability to masquerade as chronic posterior uveitis frequently leads to delayed diagnosis and inappropriate treatment, with significant prognostic implications.

**Methods:**

A narrative review was performed following a comprehensive search of the PubMed/MEDLINE and Cochrane Library databases, encompassing publications from 2000 through 2025 and including randomized controlled trials, observational studies, prospective and retrospective studies, and systematic reviews. The search strategy incorporated the following keywords: “primary vitreoretinal lymphoma”, “intraocular lymphoma”, “diagnostic vitrectomy”, “MYD88 mutation”, “IL-10/IL-6 ratio”, “intravitreal methotrexate”, “rituximab”, “CNS involvement”, “optical coherence tomography”. Two authors independently conducted study selection and eligibility assessment.

**Results:**

Heightened clinical awareness remains pivotal for diagnosing PVRL, particularly when vitritis is partially responsive to steroids, when involvement is bilateral or asymmetric, and when subretinal pigment epithelial infiltrates are present. Multimodal imaging - including optical coherence tomography (OCT), fundus autofluorescence (FAF), and fluorescein angiography (FA) - facilitates early recognition and supports clinical suspicion. Diagnostic vitrectomy with cytology, immunohistochemistry, cytokine analysis (IL-10/IL-6 ratio), and molecular testing (IgH rearrangement, MYD88 L265P mutation) significantly increases diagnostic accuracy. Due to the strong association with central nervous system (CNS) involvement, neuroimaging is mandatory at diagnosis and during follow-up. Treatment options include intravitreal methotrexate and/or rituximab, systemic high-dose methotrexate-based chemotherapy, and radiotherapy. Emerging therapies in PVRL focus on targeted agents, including intravitreal biologics, BTK inhibitors, and personalized molecular-based treatments. The optimal strategy remains controversial and should be individualized based on CNS status and patient characteristics.

**Discussion:**

Primary vitreoretinal lymphoma remains a diagnostic challenge due to its ability to mimic chronic inflammatory eye disorders and the frequent delay in establishing a definitive diagnosis. The integration of multimodal imaging, vitreous cytology, immunohistochemistry, cytokine profiling, and molecular testing has significantly improved diagnostic accuracy. However, management remains complex because of the close association with CNS involvement and the absence of universally accepted treatment protocols. Current therapeutic approaches, including intravitreal chemotherapy, systemic methotrexate-based regimens, and targeted therapies, require individualized selection based on disease extension and patient characteristics. Future advances in molecular diagnostics and personalized treatment strategies may contribute to earlier detection, improved disease control, and better long-term outcomes.

**Conclusions:**

Prompt diagnosis, combined with a structured diagnostic approach, is essential to improving outcomes in PVRL. Close collaboration among ophthalmologists, hematologists, neurologists, and pathologists is mandatory for optimal patient management.

## Introduction

Primary vitreoretinal lymphoma (PVRL) is a rare and aggressive intraocular malignancy that arises within the retina and vitreous without initial brain involvement and represents a distinct subgroup of primary central nervous system lymphoma (PCNSL) [1,2]. Despite its classification within the spectrum of PCNSL, PVRL frequently presents first with isolated ocular manifestations, making early recognition particularly challenging. Because its clinical features closely resemble chronic inflammatory eye diseases, especially uveitis, patients are often initially treated with corticosteroids, which may produce temporary improvement and further delay the diagnosis [3,4]. This diagnostic difficulty is compounded by the disease’s typically indolent early course, which may obscure its malignant nature.

Although the initial presentation may appear relatively mild, PVRL is a potentially devastating condition that can lead to irreversible visual impairment and is strongly associated with subsequent or concurrent central nervous system (CNS) involvement. The prognosis remains guarded, particularly in patients who develop CNS disease, emphasizing the importance of early detection and appropriate management [5]. The rarity of PVRL, combined with its variable clinical manifestations and overlapping features with inflammatory disorders, creates significant diagnostic and therapeutic challenges for ophthalmologists, hematologists, and neurologists alike. Establishing a timely and accurate diagnosis often requires a high index of suspicion and a multidisciplinary approach.

Advances in diagnostic techniques have improved the ability to detect PVRL at earlier stages. In addition to cytological examination of ocular specimens, modern diagnostic strategies increasingly rely on immunohistochemical, molecular, and biomarker-based analyses, including cytokine profiling and genetic testing, which enhance diagnostic sensitivity and specificity [6]. Nevertheless, obtaining adequate intraocular samples and interpreting results remains technically demanding, and false-negative findings are not uncommon.

Management of PVRL is equally complex and remains a subject of ongoing debate. Therapeutic approaches range from local ocular treatments, such as intravitreal chemotherapy or ocular radiotherapy, to systemic regimens based on high-dose methotrexate, with or without intensive chemotherapy and autologous stem cell transplantation [4]. Treatment decisions must balance effective disease control against the risks of ocular and systemic toxicity, while also accounting for the high likelihood of CNS involvement.

This review aims to provide a practical clinical approach to the diagnostic and therapeutic challenges of PVRL. Emphasis is placed on clinical presentation, key diagnostic clues, current investigative methods, and available treatment strategies to facilitate earlier recognition and improve patient outcomes.

## Methods

This narrative review encompassed a broad spectrum of study designs, including randomized controlled trials (RCTs), observational studies (cohort, case-control, and cross-sectional), prospective and retrospective studies, as well as relevant systematic reviews. Only articles published in English between 2000 and 2025 were deemed eligible. A comprehensive search of electronic databases, including PubMed/MEDLINE and the Cochrane Library, was performed. The search strategy incorporated the following keywords: “primary vitreoretinal lymphoma”, “intraocular lymphoma”, “diagnostic vitrectomy”, “MYD88 mutation”, “IL-10/IL-6 ratio”, “intravitreal methotrexate”, “rituximab”, “CNS involvement”, and “optical coherence tomography” with adaptations tailored to the indexing systems of each database. In addition, reference lists of the selected articles were manually screened to identify further eligible studies. Two independent authors screened titles and abstracts retrieved through the search, followed by full-text evaluation according to predefined inclusion and exclusion criteria. The methodological quality of the included studies was assessed qualitatively, considering study design, sample size, risk of bias, and relevance to the review objective, to contextualize the strength and reliability of the available evidence. Any discrepancies were resolved through discussion or consultation with a third author.

## Results

### Clinical presentation and diagnostic clues

Primary vitreoretinal lymphoma typically has an insidious onset with nonspecific ocular complaints, such as blurred vision, decreased visual acuity, and floaters, which often lead to delayed diagnosis [1]. The disease often resembles chronic inflammatory eye conditions and is therefore considered a masquerade syndrome. Although ocular involvement is usually bilateral, early manifestations may be asymmetric and may initially appear unilateral. Because symptoms can persist for months or even years before the correct diagnosis is established, persistent or atypical ocular inflammation should raise clinical suspicion.

Primary vitreoretinal lymphoma commonly mimics chronic posterior uveitis, and patients are often initially treated with corticosteroids for presumed inflammatory disease, which may temporarily reduce symptoms but obscure the underlying malignancy. Clinical findings that may suggest PVRL include persistent vitritis with limited anterior segment inflammation, the presence of large, atypical cells in the vitreous, and vitreous haze that does not resolve with standard therapy. Additional clues include subretinal or subretinal pigment epithelium deposits, perivascular infiltration resembling retinal vasculitis, and progressive retinal involvement [2].

Certain features may further support the diagnosis, including retinal vascular complications, macular edema, or serous retinal detachment that cannot be fully explained by inflammatory disease. The coexistence of neurological symptoms should strongly raise suspicion, as CNS involvement may occur before, during, or after ocular manifestations. For this reason, PVRL should be considered in patients with unexplained uveitis, particularly when clinical findings are atypical or when the response to conventional treatment is incomplete or short-lived [3].

### Diagnostic work-up: a stepwise approach

#### Multimodal Imaging

ultimodal retinal imaging plays a central role in the evaluation of PVRL, providing important diagnostic clues and facilitating disease monitoring. Because the clinical presentation often mimics inflammatory or infectious ocular conditions, imaging techniques are particularly valuable in raising suspicion for lymphoma and guiding further investigations. A combination of fundus photography, fundus autofluorescence (FAF), optical coherence tomography (OCT), and angiographic studies allows a more comprehensive assessment of retinal and subretinal involvement [2].

**Fig. 1:**
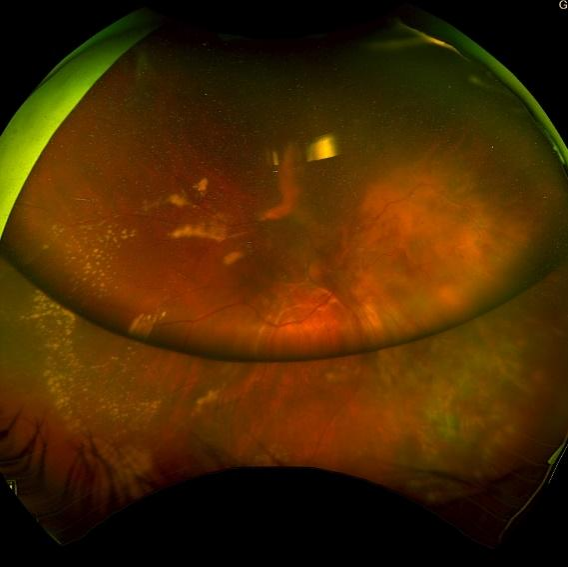
Status post diagnostic vitrectomy. Ultra-widefield image showing a superior silicone oil bubble, diffuse subretinal infiltrates in the temporal quadrant, along with a nasal subretinal infiltrate

Color fundus photography commonly demonstrates vitreous haze or cellular aggregates, optic disc edema, perivascular sheathing, subretinal pigment epithelium (sub-RPE) abnormalities, cream-colored lesions, and serous retinal detachments [7]. Several characteristic patterns have been described, including isolated vitritis, subretinal infiltrates, optic nerve involvement, and retinal detachment. Ultra-widefield imaging may reveal peripheral abnormalities that are not visible with standard fundus photography and is useful for documenting disease progression and treatment esponse (Fig. 1) [8]. In some cases, imaging can also reveal atypical presentations, including retinitis-like lesions, which should raise suspicion for PVRL when there is poor response to antimicrobial therapy [9].

Fundus autofluorescence is particularly useful for detecting RPE dysfunction and subretinal infiltration [10]. Active disease often produces areas of increased autofluorescence corresponding to sub-RPE deposits, along with mixed patterns of hyper- and hypo-autofluorescence in the macular region. Hyperautofluorescent lesions are thought to reflect altered RPE metabolism due to lymphomatous infiltration. In contrast, hypoautofluorescence may result from blockage of the normal RPE signal by tumor cells or from RPE atrophy following lesion regression [10]. Although FAF is sensitive in detecting diffuse or focal RPE abnormalities, the variability of autofluorescence patterns limits its value as a sole indicator of disease activity [11].

**Fig. 2:**
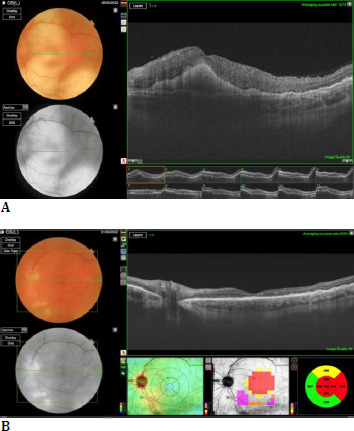
Clinical and imaging features of a patient with a diagnosis of PVRL. A. OCT showing multiple subretinal white-yellow lesions, including subfoveal (at the initial presentation). B. OCT showing the evolution after a series of intravitreal methotrexate injections

Optical coherence tomography provides detailed cross-sectional imaging and is one of the most informative tools in suspected PVRL [2]. Typical findings include vitreous cells, nodular RPE irregularities, and hyperreflective infiltrates within the outer retina or subretinal space. Additional features may include pigment epithelial detachments, epiretinal membranes, intraretinal fluid, and structural disorganization of the retina [8,12,13]. Certain OCT characteristics, such as hyperreflective material between the RPE and Bruch’s membrane or confluent subretinal bands, are considered highly suggestive of PVRL and may help distinguish it from other intraocular lymphomas (Fig. 2A) [13]. A recently described feature, vertical hyperreflective columns extending from the inner retina toward the RPE, is believed to represent microscopic tumor infiltration [14]. Optical coherence tomography is also particularly valuable for monitoring treatment response, as infiltrative lesions may regress or evolve into fibrotic scars with associated retinal thinning [2] (Fig. 2B).

Fluorescein angiography (FA) highlights RPE abnormalities and typically reveals granular or mottled patterns of hyperfluorescence and hypofluorescence [15]. These changes reflect sub-RPE tumor infiltration and may occur even in areas without obvious clinical abnormalities. Pigment epithelial detachments may show either hyperfluorescence or hypofluorescence depending on the density and viability of tumor cells. Although findings commonly associated with inflammatory uveitis, such as marked vascular leakage or cystoid macular edema, may occur in PVRL, they are generally less prominent. Angiographic abnormalities may improve with treatment and reappear with disease recurrence, making FA useful for longitudinal follow-up [16].

Indocyanine green angiography (ICGA) most frequently demonstrates hypocyanescent lesions corresponding to areas of chorioretinal damage or tumor infiltration [17]. These areas often correlate with abnormalities observed on fundus photography, FAF, and FA. However, ICGA generally provides limited additional diagnostic information compared with other imaging modalities and is primarily useful for excluding alternative diagnoses, such as inflammatory chorioretinal disorders or white-dot syndromes [2,8]. Overall, multimodal imaging represents an essential component of the diagnostic workup and follow-up of PVRL, complementing clinical examination and laboratory testing while improving recognition of this challenging disease [2].

#### Diagnostic Vitrectomy

Vitreous biopsy is considered the diagnostic gold standard for PVRL. When the diagnosis remains uncertain, additional procedures such as chorioretinal biopsy or subretinal fluid aspiration may be performed either concurrently or at a later stage [5]. In certain situations, obtaining an adequate sample may also require aqueous humor aspiration, particularly in cases of pseudohypopyon. In very rare circumstances, diagnostic enucleation may be required [18]. Close coordination between the surgical and pathology teams before the procedure is essential to ensure appropriate handling, transport media, and specimen preservation. Collected samples can be analyzed using multiple techniques, including cytology, histopathology, immunocytochemistry, flow cytometry, molecular testing, and cytokine analysis [19].

Cytological specimens should ideally be processed within one hour of collection to preserve cellular integrity. If transport delays are anticipated, the sample should be placed in a culture medium or a mild fixative solution to maintain cell viability [20]. Typically, vitreous samples are prepared on slides using cytospin methods and stained for morphological evaluation, while additional unstained preparations are reserved for immunocytochemical studies [7]. In cases with sufficient cellular material, the cell-block technique—using diluted vitreous embedded in agar or paraffin—may be applied and has demonstrated high sensitivity with low false-positive rates, particularly in distinguishing vitreoretinal lymphoma from inflammatory conditions such as idiopathic uveitis [21].

Vitreous samples may be obtained either through vitreous aspiration or pars plana vitrectomy, the latter generally being preferred because of its higher diagnostic yield [5,7]. Since lymphoma cells are fragile and prone to degeneration, corticosteroid therapy should be discontinued for at least 2 weeks before biopsy, whenever possible, to reduce the risk of false-negative results. Small-gauge transconjunctival vitrectomy techniques are commonly used and have proven both safe and effective. Retinal biopsy is rarely necessary, but when performed, tissue should be taken from deeper portions of suspicious lesions where viable tumor cells are more likely to be present [18]. Potential complications include tumor cell dissemination through surgical entry sites, and in some cases, repeated biopsies may be required, particularly when specimens contain few cells or show extensive necrosis [22].

#### Cytokine and Molecular Analysis

Histopathological confirmation of PVRL relies on conventional staining techniques such as Giemsa, hematoxylin-eosin, or Diff-Quick [23]. Cytological preparations typically reveal medium-to large-sized atypical lymphoid cells characterized by irregular nuclei, prominent nucleoli, basophilic cytoplasm, and a high nuclear-to-cytoplasmic ratio, with mitotic figures only occasionally observed [7]. Interpretation may be challenging because inflammatory cells—including small reactive lymphocytes, macrophages, and lytic cells—often predominate, obscuring the malignant population. In addition, reactive atypical monocytes may occur in non-neoplastic conditions, making immunocytochemical characterization essential. Markers of the B-cell lineage (such as CD20, CD79a, and PAX5) and the T-cell lineage (including CD2 and CD3), together with macrophage markers such as CD68, are commonly used in the diagnostic workup [24,25]. PAX5 staining is particularly useful because it can identify lymphoma cells even when membrane integrity is disrupted, and CD20 expression is difficult to detect [25,26]. Cytological analysis alone establishes the diagnosis in approximately half of cases, with relatively low rates of false-positive results [25].

Demonstration of lymphoid monoclonality significantly strengthens the diagnosis. Monoclonal B-cell populations are typically identified by restriction of immunoglobulin light chains, with either κ or λ predominance, although rare T-cell or anaplastic variants have been described [27]. Multicolor flow cytometry provides additional diagnostic information by allowing simultaneous analysis of multiple surface markers and facilitating the distinction between lymphoma and inflammatory conditions. Assessment of light-chain ratios is particularly helpful, with marked deviation from normal κ:λ ratios indicating clonality. Flow cytometry can also evaluate T-cell subsets through multiparameter gating strategies [28,29].

Molecular testing has become an important adjunct in the diagnosis of PVRL. Polymerase chain reaction–based techniques can detect clonal rearrangements of immunoglobulin or T-cell receptor genes, thereby confirming the presence of a neoplastic lymphoid population [30-32]. The sensitivity of clonality testing varies with technical factors such as primer selection, laboratory expertise, and specimen quality, and both false-positive and false-negative results may occur. Consequently, molecular findings must always be interpreted in conjunction with clinical and cytological data. Analysis of mutations in genes such as MYD88 has emerged as a useful complementary approach, particularly in samples with limited DNA content. Although MYD88 mutations are frequently detected in PVRL, their absence does not exclude the diagnosis [33].

More recently, next-generation sequencing techniques have expanded the molecular characterization of PVRL and may be particularly valuable when sample volume or cellularity is limited. These methods can identify a range of genetic alterations, some of which may have therapeutic implications and may help overcome limitations associated with traditional diagnostic techniques. Molecular profiling using intraocular liquid biopsy samples has proven feasible without compromising the material required for cytological evaluation and may improve diagnostic accuracy in challenging cases [34,35].

Measurement of intraocular cytokine levels represents another useful adjunctive diagnostic tool [7,36,37]. Elevated concentrations of interleukin-10 in aqueous or vitreous humor, particularly when the IL-10 to IL-6 ratio exceeds unity, strongly support the diagnosis of PVRL. Although threshold values vary across laboratories, elevated IL-10 levels demonstrate high sensitivity and specificity in distinguishing lymphoma from inflammatory uveitis [38,39]. Cytokine analysis may also help monitor treatment response and detect disease recurrence [37].

### Ocular and central nervous system association

Primary intraocular lymphoma, now more commonly referred to as PVRL, is strongly associated with PCNSL and rarely spreads beyond the CNS [19]. Because of this close relationship, patients suspected of having intraocular lymphoma require a thorough neurologic evaluation, including neuroimaging of the brain and orbits as well as examination of the cerebrospinal fluid (CSF) through lumbar puncture. Neuroimaging plays a critical role in detecting associated CNS disease. On computed tomography (CT), PCNSL lesions typically appear as isodense or hyperdense masses. In contrast, magnetic resonance imaging (MRI) generally demonstrates lesions that are hypointense on T1-weighted sequences and relatively hypointense or variable on T2-weighted images [40]. At the time of diagnosis, approximately 70% of CNS lesions are solitary; however, as the disease progresses, they frequently become multifocal. These lesions most commonly arise in deep cerebral structures, particularly the basal ganglia, corpus callosum, and periventricular or subependymal regions [19].

Although neuroimaging is essential for identifying CNS involvement in PCNSL, its diagnostic utility in PVRL is comparatively limited. Ocular imaging findings are often subtle or nonspecific. In a small series of patients with biopsy-proven PVRL, only a subset showed detectable ocular abnormalities on contrast-enhanced CT or MRI. Moreover, these modalities lack sufficient specificity to reliably differentiate intraocular lymphoma from other inflammatory or neoplastic conditions, such as chronic uveitis or intraocular melanoma. Consequently, ophthalmic imaging alone cannot establish a definitive diagnosis [41].

Evaluation of the CSF is another important component of the diagnostic work-up. Cerebrospinal fluid obtained by lumbar puncture should undergo cytologic examination, biochemical testing, and cytokine analysis. Malignant lymphoid cells can be detected in the CSF in roughly one-quarter of patients with brain lesions visible on MRI. Identification of lymphoma cells within the CSF confirms CNS involvement. It can establish the diagnosis of PCNSL, potentially eliminating the need for an intraocular biopsy in patients with suspected ocular disease. However, negative CSF cytology does not rule out CNS lymphoma, as the test’s sensitivity is limited. When neuroimaging reveals suspicious intracranial lesions, but CSF findings are inconclusive, a stereotactic brain biopsy may be required to obtain definitive histopathologic confirmation. In patients with no evidence of CNS disease on imaging or CSF analysis, ocular tissue sampling is necessary. In such cases, a diagnostic pars plana vitrectomy is typically performed, usually in the eye with the most pronounced vitreous inflammation or the poorest visual acuity. The strong bidirectional relationship between PVRL and PCNSL underscores the importance of long-term neurologic surveillance, as ocular disease may precede CNS lymphoma by months or years, and, conversely, CNS lymphoma may develop later in patients initially presenting with isolated ocular involvement [19,42].

### Therapeutic strategies

#### Intravitreal Therapy

Radiotherapy was historically used as a primary treatment for patients with isolated ocular involvement in intraocular lymphoma. However, its use has declined because of radiation-related ocular complications and the limitation that radiotherapy generally cannot be safely repeated if the disease recurs. For these reasons, intravitreal therapy has become a more attractive option for both primary and recurrent ocular disease. Intravitreal administration of Methotrexate (MTX) provides pharmacokinetic advantages over systemic therapy. While intravenously administered MTX maintains cytotoxic concentrations for only a few hours, intravitreal injection allows therapeutic drug levels to persist within the vitreous humor for up to five days, resulting in more sustained local tumor control [43].

The initial clinical use of intravitreal MTX was reported in a small group of four patients who received it as an adjunct to ocular radiotherapy and intensified systemic chemotherapy that incorporated blood-brain barrier disruption using intra-arterial mannitol [44]. In this early experience, all treated eyes achieved remission of intraocular disease, with follow-up periods ranging from 9 to 19 months. Subsequently, a larger clinical study evaluated intravitreal MTX in 26 eyes of 16 patients using a structured treatment protocol. The regimen consisted of an induction phase with twice-weekly intravitreal injections of 400 μg MTX for 1 month, followed by weekly consolidation injections for an additional month, and then monthly maintenance injections for 1 year [45]. Half of the patients initially presented with brain involvement related to PCNSL, whereas the other half presented with primary ocular disease. The follow-up period ranged from 6 to 35 months, with a median of 18.5 months. In four eyes, diagnostic vitrectomy alone removed the detectable tumor burden, while in the remaining eyes, malignant cells disappeared after approximately 12 injections. Disease recurrence occurred in three patients, but remission was again achieved after a second course of intravitreal MTX therapy. The most frequently reported adverse effects included cataract formation, corneal epitheliopathy, and maculopathy. Less common complications included limbal stem cell deficiency, optic nerve atrophy, and sterile endophthalmitis [19,45].

Intravitreal MTX may also be useful in cases of recurrent intraocular lymphoma. For example, one patient with relapsing disease after radiotherapy and multiple systemic chemotherapy regimens was treated with repeated intravitreal injections of MTX combined with Thiotepa, resulting in complete tumor regression that persisted for at least 156 days. Although intravitreal therapy offers the advantage of avoiding systemic toxicity while maintaining consistent therapeutic concentrations within the eye, it does not address lymphoma involvement within the CNS or the contralateral eye [19].

Intravitreal administration of Rituximab is another therapeutic option for the management of intraocular lymphoma. The commonly used dose is 1 mg in 0.1 mL, delivered directly into the vitreous cavity [46]. Rituximab is a monoclonal antibody directed against the CD20 antigen expressed on B lymphocytes, making it particularly useful in the treatment of B-cell lymphomas. Currently, there is no universally accepted treatment protocol for intravitreal rituximab. Reported regimens vary considerably, ranging from a single injection to repeated injections administered at different intervals [47]. In clinical practice, however, injections are most often given at approximately four-week intervals. Initial therapeutic responses are generally favorable, with many patients showing regression of intraocular lymphoma. Nevertheless, relapses are relatively common, and recurrent disease may require additional treatment with intravitreal MTX or ocular radiotherapy [46]. Although intravitreal rituximab is generally well tolerated, several ocular complications have been reported following treatment. These include cataract formation, elevation of intraocular pressure, granulomatous anterior uveitis, vitreous hemorrhage, and retinal detachment. Careful monitoring is therefore required during treatment to detect and manage potential adverse effects [47].

Despite advances in local and systemic therapies, the optimal treatment strategy for PVRL or for PCNSL with ocular involvement remains uncertain. Establishing standardized treatment guidelines is challenging because these malignancies are rare. Most of the available evidence comes from small case series or retrospective reports, making it difficult to directly compare different treatment approaches. Moreover, large randomized clinical trials comparing combined radiotherapy and chemotherapy with chemotherapy alone are often impractical or ethically complex. As a result, findings from studies of PCNSL cannot always be directly applied to patients with isolated intraocular disease, further complicating treatment decisions [19,48].

#### Systemic Chemotherapy

Systemic chemotherapy is a fundamental component of PVRL management, particularly when there is concurrent or suspected CNS involvement. It may be used either as a primary treatment modality or in combination with radiotherapy. However, the effectiveness of systemic therapy is limited by physiological barriers such as the blood-brain and blood-ocular barriers, which restrict the penetration of many chemotherapeutic agents into the retina, vitreous, and CNS. Therefore, treatment protocols typically employ drugs capable of crossing these barriers, most notably MTX and cytosine arabinoside (cytarabine). Systemic chemotherapy can be administered intravenously or intrathecally, with intrathecal therapy particularly useful in patients with CNS disease or leptomeningeal involvement [48].

High-dose MTX is considered the backbone of systemic chemotherapy for PVRL and for PCNSL, as it can achieve therapeutic levels within both the CNS and ocular tissues. When administered at high doses, MTX can penetrate the blood-brain barrier and has demonstrated complete response rates ranging from approximately 50% to 80% [49]. In most treatment protocols, MTX is administered intravenously at a dose of around 8 g/m^2^. Therapy typically consists of an induction phase in which the drug is administered every 14 days until a complete response is achieved, followed by a consolidation phase of two additional doses given at the same interval. Maintenance therapy is then continued at 28-day intervals for approximately 11 months to reduce the risk of relapse [50]. In certain cases, MTX is also administered intrathecally, usually at doses of 12 mg (or 6 mg in selected protocols), either through lumbar puncture or via an Ommaya reservoir to allow repeated access to the CSF. Despite its therapeutic benefits, systemic MTX may cause several ocular adverse effects, including periorbital edema, excessive lacrimation, photophobia, blepharitis, and conjunctival hyperemia. Intrathecal MTX is particularly valuable in PVRL cases associated with CNS involvement [51].

Cytosine arabinoside (cytarabine, Ara-C) is another chemotherapeutic agent frequently incorporated into systemic treatment regimens for PVRL because of its ability to penetrate the blood-ocular barrier [52]. High-dose cytarabine is typically administered intravenously at 2-3 g/m^2^ over multiple cycles, often spanning a three-month period with approximately six treatment cycles [53]. Like MTX, cytarabine may also be administered intrathecally at a dose of about 100 mg per cycle to target malignant cells within the CSF [51]. Although effective, cytarabine therapy may be associated with ocular complications, including ocular irritation, conjunctivitis, and keratitis, and prophylactic topical corticosteroids are often used to minimize corneal toxicity during treatment [48].

In addition to conventional chemotherapeutic agents, targeted immunotherapy with rituximab has become an important adjunct in the treatment of PVRL [51]. Intravenous rituximab is commonly administered in combination with MTX-based chemotherapy, particularly in cases of recurrent or relapsed disease [46,51]. The typical systemic dose is 375 mg/m^2^, and the drug enhances tumor cell destruction through immune-mediated mechanisms, including complement activation and antibody-dependent cellular cytotoxicity [48].

Combination chemotherapy regimens have been increasingly employed to improve therapeutic outcomes and reduce relapse rates. For instance, the combination of MTX and cytarabine has demonstrated promising results in patients with both ocular and CNS involvement, with studies reporting disease resolution in both compartments and remission durations exceeding 24 months [54]. More intensive multi-agent regimens have also been explored, such as the MATRix protocol, which includes MTX, cytarabine, thiotepa, and rituximab. This regimen has shown improved treatment outcomes, particularly in patients younger than 70 years, with complete remission rates of approximately 50%. In comparison, treatment with MTX and cytarabine alone has achieved remission rates of approximately 23%, whereas adding rituximab to these agents has increased them to approximately 30% [55]. These findings suggest that more aggressive combination therapy may provide superior disease control in selected patients.

For patients with refractory or recurrent PCNSL or PVRL, high-dose chemotherapy followed by autologous stem cell transplantation may be considered. This approach allows the administration of intensive chemotherapy regimens that would otherwise cause severe bone marrow suppression. Before treatment, hematopoietic stem cells are harvested from the patient and subsequently reinfused after chemotherapy to restore bone marrow function. Autologous stem cell transplantation has therefore emerged as a valuable salvage therapy for patients who fail to respond to standard treatment or who experience disease relapse [48,56].

#### Radiotherapy

External beam radiotherapy is commonly used in patients with PVRL who do not demonstrate evidence of CNS involvement [19,57]. Historically, radiation was delivered at a total dose of approximately 54 Gy. However, because of the significant risk of radiation-induced retinopathy, treatment protocols have been modified to use lower doses. Currently, external beam radiotherapy is typically administered at a total dose of 35-40 Gy, delivered in approximately 15 fractions of 2 Gy each. Radiation is usually delivered with opposed lateral beams to ensure both eyes are adequately treated, even if the disease appears unilateral, given the high likelihood of bilateral involvement [58].

In patients with CNS dissemination, whole-brain radiotherapy may be combined with ocular radiotherapy. This approach is generally reserved for cases in which the disease does not respond adequately to systemic chemotherapy or when patients are unable to tolerate aggressive systemic treatment regimens [58]. While whole-brain radiotherapy can be effective in controlling CNS lymphoma, high-dose irradiation with low fractionation schedules is associated with an increased risk of neurotoxicity, which may manifest clinically as cognitive impairment, mood disturbances, or depressive symptoms [42].

Despite its therapeutic benefits, external beam radiotherapy may result in several ocular complications. These adverse effects include radiation dermatitis, punctate keratopathy, radiation retinopathy, cataract formation, radiation-induced optic neuropathy, and dry eye syndrome. Careful dose planning and long-term ophthalmologic monitoring are therefore essential to minimize treatment-related morbidity [42].

### Prognosis and follow-up

Primary intraocular lymphoma, particularly when associated with CNS involvement, is characterized by an aggressive clinical course and generally poor prognosis. The ocular outcome is often unfavorable due to the disease’s malignant nature and its strong tendency to involve the CNS. Reported mortality rates in PVRL vary widely, ranging from approximately 9% to 81% over 12- to 35-month follow-up periods [59,60]. Early diagnosis appears to significantly influence survival outcomes. Studies have shown that patients diagnosed with PVRL before the development of CNS disease have a longer survival, with an average of about 60 months compared with approximately 35 months when CNS involvement is detected later. Prognostication in cases with CNS involvement may be assessed using the International Extranodal Lymphoma Study Group scoring system, which is primarily designed for PCNSL and applies to PVRL only when CNS disease is present [55]. Overall, PCNSL carries a poor prognosis, with reported five-year survival rates of around 30% [61]. Treatment outcomes vary depending on the therapeutic approach: patients treated with radiotherapy alone or combined chemoradiotherapy have a median survival of roughly 10-16 months, whereas MTX-based chemotherapy regimens, sometimes combined with agents such as ifosfamide or trofosfamide, can extend median survival to approximately 30 months. In addition, clinical studies have demonstrated that despite the use of multiple treatment modalities, including systemic and ocular therapies, overall survival remains limited. For example, one study reported a median overall survival of about 31 months and a median progression-free survival of 18 months, with ocular-directed therapy not significantly improving overall survival outcomes [62,63].

Follow-up in PVRL is essential because the disease has a high risk of recurrence and progression to the CNS. Long-term monitoring involves both ophthalmologic and neurologic evaluation to detect local relapse or systemic dissemination at an early stage. Patients are typically reviewed every 3-4 months during the first two years after treatment, when the risk of recurrence is highest, and then every 6-12 months thereafter. Close collaboration between ophthalmologists, oncologists, and neurologists is important during follow-up, as early detection of recurrence allows prompt initiation of systemic or local therapy, which may improve disease control and patient outcomes [48].

### Future perspectives

Future research in PVRL is focused on improving early diagnosis, optimizing treatment strategies, and developing targeted therapies to enhance patient outcomes. Advances in molecular diagnostics have significantly improved the understanding of the disease pathogenesis. Detection of genetic mutations, particularly MYD88 L265P, as well as immunoglobulin heavy chain (IgH) gene rearrangements and cytokine profiling (such as elevated IL-10 levels), has emerged as a valuable diagnostic and prognostic tool. These molecular biomarkers may also help identify patients at higher risk of CNS involvement and guide personalized treatment approaches [64,65].

Targeted therapies represent an important emerging area in the management of intraocular lymphoma. Agents targeting the B-cell receptor signaling pathway, such as Bruton’s tyrosine kinase (BTK) inhibitors, including ibrutinib and tirabrutinib, have shown promising results in patients with relapsed or refractory disease. In addition, immunomodulatory drugs such as lenalidomide, often used in combination with rituximab, are being investigated as alternative systemic treatments. Other potential therapeutic strategies include PI3K and mTOR inhibitors, which target molecular pathways involved in lymphoma cell survival and proliferation [66].

Immunotherapy is also gaining increasing attention in the treatment of PVRL. Novel approaches such as immune checkpoint inhibitors and chimeric antigen receptor T-cell therapy targeting CD19-positive B cells may provide new options for patients with advanced or treatment-resistant disease. Furthermore, the integration of multimodal imaging techniques and artificial intelligence-based analysis may improve the early detection and monitoring of disease activity, particularly in distinguishing intraocular lymphoma from chronic uveitis [66].

Despite these promising developments, the rarity of the disease continues to limit large-scale clinical trials. Future progress will therefore depend on multicenter collaborative studies aimed at establishing standardized diagnostic criteria and treatment protocols. Advances in molecular biology and targeted therapy are expected to play a crucial role in the development of personalized medicine approaches, ultimately improving prognosis and long-term survival in patients with intraocular lymphoma [6].

## Conclusions

Primary vitreoretinal lymphoma is a rare and aggressive malignancy that poses significant diagnostic and therapeutic challenges due to its ability to mimic chronic uveitis and its strong association with CNS involvement. Early diagnosis requires a high level of clinical suspicion and the use of multimodal diagnostic techniques, including imaging, cytopathology, and molecular analysis. Current treatment strategies, such as intravitreal chemotherapy, systemic MTX-based regimens, and radiotherapy, can achieve disease control; however, recurrence remains common, and long-term prognosis is still limited. A multidisciplinary approach and close follow-up are essential for optimal management. Advances in molecular diagnostics and targeted therapies may improve future treatment strategies and outcomes for patients with this challenging disease.
